# Adolescent Emotional Maturation through Divergent Models of Brain Organization

**DOI:** 10.3389/fpsyg.2016.01263

**Published:** 2016-08-23

**Authors:** Jose V. Oron Semper, Jose I. Murillo, Javier Bernacer

**Affiliations:** Mind-Brain Group, Institute for Culture and Society, University of NavarraPamplona, Spain

**Keywords:** emotion regulation, executive functions, integration, personal identity, socialization

## Abstract

In this article we introduce the hypothesis that neuropsychological adolescent maturation, and in particular emotional management, may have opposing explanations depending on the interpretation of the assumed brain architecture, that is, whether a componential computational account (CCA) or a dynamic systems perspective (DSP) is used. According to CCA, cognitive functions are associated with the action of restricted brain regions, and this association is temporally stable; by contrast, DSP argues that cognitive functions are better explained by interactions between several brain areas, whose engagement in specific functions is temporal and context-dependent and based on neural reuse. We outline the main neurobiological facts about adolescent maturation, focusing on the neuroanatomical and neurofunctional processes associated with adolescence. We then explain the importance of emotional management in adolescent maturation. We explain the interplay between emotion and cognition under the scope of CCA and DSP, both at neural and behavioral levels. Finally, we justify why, according to CCA, emotional management is understood as regulation, specifically because the cognitive aspects of the brain are in charge of regulating emotion-related modules. However, the key word in DSP is integration, since neural information from different brain areas is integrated from the beginning of the process. Consequently, although the terms should not be conceptually confused, there is no cognition without emotion, and vice versa. Thus, emotional integration is not an independent process that just happens to the subject, but a crucial part of personal growth. Considering the importance of neuropsychological research in the development of educational and legal policies concerning adolescents, we intend to expose that the holistic view of adolescents is dependent on whether one holds the implicit or explicit interpretation of brain functioning.

## Introduction

Unraveling the structure and function of the brain is still a challenge for most psychologists and philosophers of mind. Any empirical approach is underpinned by an interpretation of brain functioning, either explicit or implicit. In this paper we argue that neuropsychological adolescent maturation may have opposing explanations depending on the interpretation of the assumed brain architecture. To prove this assertion, we will first explain the two most common accounts of brain organization. They are overtly divergent because one proposes that the brain is divided into functionally specified modules, while the other defends the idea that the brain is a dynamic complex system and that functionality emerges from the whole. Note that the former assumes some interaction between modules, although this occurs at the end of the process, that is, after each brain area-or module-has taken care of its function. Conversely, in the latter, *interaction* happens at the beginning of the process, and there is no single function where the activity of the whole brain is not relevant. Therefore, we hypothesize that the understanding of emotional management is different according to the underlying comprehension of brain functioning. This hypothesis can be subdivided into two additional sub-hypotheses: (i) if the modular view is accepted, the key fact in emotional management is the regulation of the emotional module by the cognitive module; (ii) if the systemic view is accepted, the key fact is that emotional and cognitive information is integrated at the beginning of the process. In this paper, *regulation* is understood as bringing emotion-or the neural activity of ‘emotion-related’ brain areas- under the control of cognition -or the neural activity of ‘cognition-related’ brain areas-. On the other hand, *integration* involves that cognition and emotion are continuously interacting, so there is no cognition without emotion, and vice versa.

After explaining these two interpretations in some detail, we will describe the main neurobiological facts about adolescent maturation. We will first outline the hormonal processes underlying the beginning of adolescence, and then summarize the neuroanatomical and neurofunctional changes that characterize the transition from childhood to adolescence. Next, we will highlight the importance of emotional management in adolescent development, at both a behavioral and neurobiological level. We will also develop our main claim: divergent accounts of brain functioning result in opposing interpretations of neurobiological development, and lead to different models of emotional management. In our opinion, revealing these two global interpretations, the theoretical background in which they are rooted, and their consequences is important for present and future legal and educational policies for adolescents.

## Two Accounts of Brain Functioning: Componential and Systemic

While a discrete classification of all the views of brain functioning on offer may be unfair and in any case implausible, two views are overtly divergent: the componential computational account (CCA) and the dynamical systems perspective (DSP). The former is the most deeply rooted and widespread in experimental neuroscience, while the latter has recently been applied to psychology and neuroscience. Although CCA was founded much earlier, Fodor’s (1983) theory of modularity has been very influential on its recent development. The main idea is that some cognitive functions are modular, which means that they are domain-specific, automatic, fast, and informationally encapsulated. Although this is a modular explanation of the mind, it can be directly applied to the brain in as much as modules have a concrete brain correlate. Thus, according to CCA the brain is composed of different modules that interact with those to which are anatomically or functionally coupled. Interestingly, Fodor (2000) argues that cognitive functions other than perception and language could be non-modular; although other authors defend a massive modular theory (Pinker, 1997). From a neural point of view, the most common conclusion under the scope of CCA is the association of a brain area with a particular function. In this sense, neuroimaging methods are the most common resource for supporting the strict association between a brain area and a particular function. This is why some authors have warned about the danger of using neuroimaging to promote a “new phrenology” (Poldrack, 2010). Therefore, one of the main characteristics of a CCA of the mind and brain is temporal stability, that is, the nearly permanent association between selective brain areas, as well as a stable identification of a region with a particular function.

The DSP, however, argues that the brain is a complex dynamic system rather than a group of interacting modules. This means that it “follows principles of self-organization in which global states might be characterized in terms of collective variables that follow a dynamic capable of generating enormous human behavioral complexity” (Kelso, 1995). As Anderson (2015) has stated in a recent book, the main reason why a brain is not analogous with a computer is that the former has an intrinsic dynamic, whereas the latter is inactive while waiting for the user’s order. The brain’s intrinsic dynamic is also known as the “resting state” of the brain, which is the activity pattern that the brain has “by default.” Given the relevance of this issue for the dynamic systemic view of the brain, we review the main changes in the brain’s resting state during adolescence in Section “Neurofunctional Development.” Hence, from DSP, the brain’s response to a stimulus is a deviation of its intrinsic dynamics that should be understood as a whole, even though some regions could have a tendency to be more engaged in responding to certain kinds of stimuli. This dispositional tendency is characterized by Anderson as an *interactive differentiation*, which means that brain areas tend to differentiate across development in order to perform a particular type of function. However, differentiation is not as restrictive as specialization, since the former allows a relatively fast re-acquisition of a new function when the context changes. Anderson explains interactive differentiation by reference to past research that shows a “relatively rapid crossmodal recruitment of occipital cortex to process tactile information” (Merabet et al., 2008). Moreover, Anderson (2010) defends neural reuse as the cornerstone of the functional anatomy of the brain. He explains its main characteristics and differences with respect to neural plasticity as follows: “According to neural reuse, circuits can continue to acquire new uses after an initial or original function is established; the acquisition of new uses need not involve unusual circumstances such as injury or loss of established function [as in plasticity]; and the acquisition of a new use need not involve (much) local change to circuit structure [as in plasticity] (e.g., it might involve only the establishment of functional connections to new neural partners)” (Anderson, 2010). In a similar way, Pessoa (2013) mentions “temporal assemblies.” This means that brain regions, even individual neurons, may engage in many different types of tasks. For example, it is reported that Broca’s region is activated more in non-language than in language tasks (Poldrack, 2006). Remarkably, brain functioning is understood under DSP as context-dependent, since temporal assemblies of brain areas have different constraints depending on both the internal-intrinsic dynamics-and external-particular situation of the agent-context.

In summary, according to CCA cognitive functions are associated with the activity of restricted brain regions, and this association is temporally stable; by contrast, DSP argues that cognitive functions are better explained by interactions between several brain areas, whose engagement in particular functions is context-dependent and based on neural reuse. Its main principle is the emergence of perception and behavior through the global activity of the brain (Tognoli and Kelso, 2014). From a neuroanatomical point of view, some interpretations of DSP may allow modularity and brain functional segregation (Bullmore and Sporns, 2009). However, modularity is understood here as a transient characteristic of the whole system, which fulfills the conditions of being context-dependent and relying on neural reuse (Anderson, 2010). This is different from the modular organization of the brain and mind that CCA defends, since according to the latter modules are specialized and fixed at some point of brain development. There is unquestionably some interaction between modules, but there is a crucial nuance: whereas in CCA functions are defined before the interaction (for example, a cognitive module interacts with an emotional module), in DSP functions emerge after the interaction between brain areas (for example, the interaction between the occipital cortex and the postcentral gyrus is involved in tactile discrimination in blindfolded participants). The divergence between these interpretations is clearly explained in Interlude 1 of Anderson’s book (Anderson, 2015). For the past 20 years, the trend in neuroimaging has been selectivity, i.e., demonstrating that a brain area is selectively active in a particular task. However, different tools have been developed in recent years (for example, Cognitive Neuroscience 2.0 by Yarkoni et al., 2010) to evaluate the various tasks that recruit a particular brain area, and all the reported areas that are recruited by a particular task. This type of meta-analyses reveals that the association between concrete areas and certain functions are not restricted. According to Anderson, this is an evolutionary strategy that makes complete sense: it is more effective to use and reuse different brain areas for specific immediate context-dependent purposes than developing new masses of brain tissue for emerging functions. On the other hand, many studies demonstrate that focal lesions seem to be associated with selective functional impairments. Focusing on emotion-related cases, bilateral damage in the amygdala has been linked to specific problems with fear processing (see, for example, Adolphs et al., 1994). This would support CCA and anatomical modularity. However, Anderson defends the idea that neural reuse can also account for these examples, and that this specific relationship between structure and function can be also explained from a non-modular perspective (Anderson, 2010). In fact, there are also studies on amygdala lesions that do not report impairments in fear-related behavior (Feinstein et al., 2013, for instance).

Having explained these two opposing accounts of brain functioning and architecture, in the following section we will outline the main neurobiological facts of adolescent maturation. We will then interpret these facts under the scope of CCA and DSP, and explain adolescent emotional management in the context of neurobiological development and both views of brain organization.

## Neurobiology of Adolescent Maturation: A Brief Review

### Beginning and End of Adolescent Development

Adolescent development begins due to hormonal changes. It is characterized by three events: adrenarche, gonadarche, and activation of the growth axis (Blakemore et al., 2010). These hormonal changes trigger a new configuration of the brain, which is progressively settled depending on the adolescent’s behavior. How does the process end? Although it obviously depends on biological processes as well, these can be accelerated or delayed by stress (Hensch and Bilimoria, 2012; Callaghan et al., 2013), social factors (Blakemore, 2008; Crone and Dahl, 2012), genetics, lifestyle, past experiences, and personal decisions (Blakemore, 2008; Schott et al., 2011). This means that the maturational window of adolescence may remain open in abnormal situations (Choudhury et al., 2012; Giedd, 2012). One of the main messages of this section is that development during adolescence entails a plethora of processes that interact with each other. Therefore, development should be interpreted as a global rather than a domain-related process.

### Neuroanatomical Development

Regarding neuronal and synaptic development during adolescence, the prefrontal cortex grows significantly with respect to the rest of the frontal cortex (Kanemura et al., 2014). This is of great importance, since this cortical region is fundamental in typically human behavior (Fuster, 2001; van den Heuvel and Sporns, 2011; de Reus and van den Heuvel, 2014). Adolescence is a time of synaptic pruning, when those synapses that are in use are strengthened, and those unused are lost (Huttenlocher and Dabholkar, 1997; Powell, 2006; Blakemore, 2008); therefore, the subject’s behavior is essential in neuroanatomical development. Another interesting finding is that, whereas axonal development is mostly linear during adolescence, the increase in synaptogenesis in certain aspects of the prefrontal, parietal, and temporal cortices is quadratic (Blakemore and Choudhury, 2006; Dennis and Thompson, 2013). Concerning white matter development, the myelin layer of axons is engrossed and subsequently neural communication is faster and more effective (Hartline and Colman, 2007). As happens in the case of gray matter, the development of white matter is differential depending on the brain area concerned (Dennis and Thompson, 2013). For example, the axons connecting the hippocampus and entorhinal cortex are fully myelinated by the first year of age; however, fibers between the hippocampus and the parahippocampal cortex are myelinated during adolescence (Benes, 1989; Benes et al., 1994). The functional meaning of this finding seems to be an enhanced development of semantic memory during adolescence (Vargha-Khadem et al., 1997; Ramos, 2013). The hippocampal connections with the amygdala are also strengthened (LaBar and Cabeza, 2006), contributing to the maturation of limbic circuits. There is also an important progression in the neural substrate of attention, due to the development of the fibers connecting the ventrolateral prefrontal cortex and temporal areas, in addition to the projections between the anterior cingulate, parietal, and occipital cortices (Schott et al., 2011). These attention-related processes allow the lateral prefrontal cortex to disengage from well-known tasks and to supervise novel actions (Velanova et al., 2008). Once again, considering neuroanatomical development, it seems that growth-in a broad sense-during adolescence involves mutual relationships between cognitive and emotional processes.

### Neurofunctional Development

The neuroanatomical changes mentioned above are related to the development of different functional features, such as memory, judgment, temporal discounting of rewards, cognitive interpretation of emotions, decision making, response to salient stimuli, and socialization. It is worth noting that the development of each of these processes is not independent; they are intertwined with one another. We will now summarize the main neurofunctional changes associated with these processes, emphasizing those more closely related to emotion and cognition.

In the course of adolescence, there is a significant maturation of memory. This involves, among other issues, the development of semantic memory-associated with the perirhinal cortex-away from episodic memory-associated with the hippocampus (Ofen and Shing, 2013). Interestingly, there is a complete maturation of the process of remembering, which becomes a global operation involving the whole person. In addition to memory, there is a development in the temporal devaluation of rewards. This process is an indicator of impulsivity (Anokhin et al., 2015) and is relevant for emotional management, since higher delay-discounting (i.e., short-term impatience) is associated with lower emotional stability (neuroticism; Manning et al., 2014). At a behavioral level, recent research reveals a high test–retest correlation in a delay-discounting task in adolescents, even though the measures were taken 2 years apart. Interestingly, together with this correlation, the authors found a significant decrease in temporal devaluation in the two-year interval (Anokhin et al., 2015). Moreover, there is a development of temporal discounting during adolescent maturation, since the prefrontal cortex will attenuate the hyperactivation of the nucleus accumbens in reward anticipation (Somerville and Casey, 2010). As a result, temporal delay is not such a strong devaluator of rewards in adults as it is in adolescents. In turn, van den Bos et al. (2015) recently reported that an age-related decrease in impatience was associated with an increased negative functional coupling between the right dorsolateral prefrontal cortex and the striatum. These results show that temporal discounting is an impulsivity-related trait that changes progressively during adolescence, and that these behavioral changes have a neurobiological correlate.

During adolescence, there is also a development of executive functions such as judgment. The progressive increase in activity of the ventromedial prefrontal cortex in these situations enhances control of the amygdala and insula, which boosts cognitive re-evaluation-the re-interpretation of emotions. In a similar way, limbic-related regions such as the anterior cingulate and insula show decreased activity with age-within adolescence-in stressing situations, suggesting a more sober evaluation of events (Pitskel et al., 2011). In fact, this down-regulation of the anterior cingulate and insula is more common in socially successful youngsters who turn out to be more resilient adults (Blakemore and Choudhury, 2006). These authors relate this fact with the ability to simulate and predict conflictive scenarios, thus rehearsing possible solutions. But this developmental feature not only includes limbic areas, but also ‘cognitive-related’ regions, since it is also related to the incremental association with age between the parietal and dorsolateral prefrontal cortex (Blakemore and Choudhury, 2006). This cognitive-emotional integration is also evident in the interaction between the amygdala, hippocampus, ventromedial, lateral, and dorsolateral prefrontal cortex in the maturation of objective judgment (Blakemore, 2008; Baumgartner et al., 2013; Ertl et al., 2013). Similar players are involved in the progressively increased control of personal affectation after salient stimuli. The ability to control the autonomic response in these situations, which is clearly more developed in adults than adolescents, is related to the influence of the dorsolateral prefrontal and anterior cingulate cortices on the anterior insula (Strang et al., 2011). Besides this, the interaction between the anterior cingulate cortex and the basolateral amygdala increases over the course of adolescence, correlating with a better control of anxiety (Cunningham et al., 2002).

As an additional note we would like to draw attention to the fact that, in general, all these tasks require an activation of the prefrontal cortex in adolescents, whereas in adults this activation is located in most posterior-parietal or temporal-regions. As a consequence, the prefrontal cortex is available for demanding cognitive tasks in adults, which can be considered an acquired disposition for successful performance (Bernacer and Murillo, 2014; Orón, 2014).

Finally, we will briefly summarize the main changes that occur in the resting state of the brain during adolescence. As Johnson (2011) wrote, “while we have some good examples of functional brain development at the level of individual cortical regions, we are still largely in the dark about how the larger scale of cortical function in terms of networks of regions develops.” Johnson cites the work of Fair et al. (2007, 2009) to summarize the main differences in resting state between adolescents and adults: a decreased short-range connectivity, and an increased long-range connectivity. Anderson explains this in terms of his *interactive differentiation and search*: both phenomena allow the configuration of transient neural assemblies involving proximal and distal groups of neurons, as well as increasing the scope of tasks with which these groups may be associated (Anderson, 2015). More recent work shows that the main functional networks of the brain are fully organized during childhood, while functional coupling between networks is developed during adolescence (Marek et al., 2015). This progressive maturation of the brain is related, according to the authors, to cognitive control (the ability to execute goal-directed behavior).

These are the main neurobiological events that affect youngsters during adolescence. This review, although short, is thorough. Our main intention is to demonstrate that adolescent neurodevelopment is not restricted to a particular set of brain areas, but is a rather global process that involves all types of neural information-cognitive, sensorimotor and limbic. Whole-brain changes seem to be necessary for an adequate maturation. However, these neurobiological facts may have a different interpretation in DSP and CCA: according to the former, all changes happen as dynamic interrelated processes, whereas the latter defends a domain-related maturation and a subsequent relation between domains. In the next section, we will discuss the importance of emotional management in adolescent maturation, as it has been highlighted by several currents in psychology. Furthermore, we will explain how emotional management is viewed from CCA and DSP, giving rise to very different interpretations.

## Adolescent Development and Emotional Management

Each stage in life brings its own challenges: moving forward or bearing one’s own maturational deficiencies is at stake (Marcia and Josselson, 2013). This view of a gradual development with identifiable stages can be found in important theories such as Piaget’s (1974), on cognition, Kohlberg’s (1973), on morality, and Erikson’s (1998), on global personal development. Adolescent maturation as a stage in the development of personal identity has been also studied from a neuropsychological perspective (Meeus et al., 2002; Sebastian et al., 2008; Kroger et al., 2010; Crone and Dahl, 2012; Konrad et al., 2013; Luyckx et al., 2013). Therefore, all the neuroanatomical processes that occur during adolescence should be understood in the context of the global development of personal identity. Although identity maturation does not start in adolescence, there is an initial re-elaboration of identity during this period of life. Besides this, there are two other maturational processes of special importance in adolescence: socialization and the development of executive functions. These three elements can be conceptualized as different, but they should be considered intimately integrated in order to understand the reality of youngsters. Emotional management is unquestionably a necessary condition for an adequate development of personal identity (McLean, 2005), socialization (Eisenberg, 2000), and executive functions (Hofmann et al., 2012). However, it can be interpreted differently depending on the account of brain functioning that one assumes. Thus, in this section we will summarize the impact of understanding the brain as a set of modules or as a dynamic system on emotional management. As mentioned above, the CCA of emotional management is predominant in most empirical or theoretical studies; for that reason, the explanation from DSP will be more speculative.

### Adolescent Emotional Management and the Componential Computational Account

The mainstream view of the neuropsychological development of adolescents leans on emotional self-regulation and the control of risky behaviors, either intrinsic or extrinsic (Vohs and Baumeister, 2011; Smith et al., 2013; Bjork and Pardini, 2014; Goesling et al., 2014; Gross, 2014; Noël, 2014; Willoughby et al., 2014). This view is supported by the discovery of an earlier maturation of the basal ganglia with respect to prefrontal aspects of the cortex, and an overall development of bottom-up networks prior to the consolidation of top-down connections (Rubia et al., 2006; Rubia, 2013). At a behavioral level, sensation-seeking through adolescence follows an inverted-U shape, whereas impulsivity and self-control follow a linear decrease and increase, respectively (Steinberg, 2010). Thus, the general view of adolescence in neuropsychology is some sort of brute force that is out of control; and different policies to promote its extrinsic or intrinsic regulation, or even to avoid any kind of regulation, are proposed (Steinberg, 2008).

In our opinion, this widely accepted view of emotional management is based on CCA: there are different mind (or brain) modules and, in a “normal” (adult) condition, some (cognitive modules) regulate others (emotional modules). Hence, the key word in emotional management under the scope of CCA is regulation. In turn, a recent article summarizes the neural bases of emotional regulation, and its inspiration is clearly componential computational (Etkin et al., 2015). According to these authors, emotions are determinants of behavior and thought, and they have to be regulated; otherwise they can be excessive or insufficient. They define emotions, following Mauss et al. (2005), as “sets of cognitive, subjective, physiological, and motor changes that arise from an individual’s conscious or non-conscious determination that a stimulus has a positive or negative value in a particular context and with respect to that individual’s currently active goals.” The reason why they can be excessive or insufficient is that their components (cognitive, physiological, motor, etc.) may be discordant in terms of timing, magnitude, and duration. In general, emotion regulation is based on the differential functional attributed to “cognitive” and “emotional” brain regions, and the regulation of the latter by the former. Indeed, subcortical nuclei such as the amygdala and nucleus accumbens develop early in adolescence, due to hormonal activity, thus changing the world of expectations and motivations of youngsters (Spear, 2000; Mills et al., 2014). In addition to this, subcortical-cortical connections from these nuclei to the prefrontal cortex mature earlier than regulatory prefrontal cortico-subcortical projections (Somerville and Casey, 2010), initiating a period of special vulnerability in adolescence (Casey et al., 2011) that may end quite late in adulthood (at about 25–30 years of age; Steinberg, 2008). In a similar way, other authors suggest a triadic model where the nucleus accumbens, amygdala, and prefrontal cortex would be related to reward-seeking, harm-avoidance, and supervision, respectively (Ernst et al., 2006; Ernst and Fudge, 2009). The triadic account can also be understood as an interplay between social (linked to temporal cortical areas), personal-emotional (linked to subcortical nuclei), and cognitive (linked to prefrontal cortex) aspects of adolescence (Nelson et al., 2005). According to Etkin et al. (2015), emotions may be regulated through two different mechanisms, namely explicit and implicit. The former is linked to consciousness, and the brain areas involved in the process are cortical (ventrolateral and dorsolateral prefrontal, parietal and supplementary motor cortex); the latter involves an unconscious self-regulation of the brain, carried out by the accumbens and ventromedial prefrontal cortex.

Interestingly, the main authors that suggested neuro-developmental models of adolescence in the last few decades have recently published updated versions of their proposals. One of the most popular is the dual systems model offered by Steinberg, which is based on the differential developmental pace of the striatum and ventromedial prefrontal cortex, with respect to that of the lateral prefrontal, parietal, and cingulate cortices (Shulman et al., 2016). Although the authors admit that the model is an oversimplification, they defend its validity for explaining some attitudes of adolescents, such as risk-taking behavior. Further, some triadic models also include a third system, governed by the amygdala, which would be responsible for emotional intensity and avoidance (Ernst, 2014). Another version of the triadic model has been proposed by Nelson et al. (2016). According to them, the driving force of the adolescent’s development is social behavior, which modulates subcortical structures (thus having an impact on affect-driven motivation) that interact with various cortical regions (modulating sensory and perceptual social representations and executive functions; Nelson et al., 2016). Overall, in our opinion, all these proposals are based on an interpretation of the brain that identifies structure and function, no matter how many physical and/or mental modules are included in the model. Other prestigious authors on the topic propose a change in the interpretation of adolescent development, moving to a more integrated account that they term the “imbalance” model (Casey et al., 2015). This looks beyond the modular additive model, and considers behavioral development under the scope of distributed interactive networks, since the brain correlates of adolescent behavior are not orthogonal, but interactive and integrative. They also state the following: “Perhaps the most prominent difference between dual systems and imbalance models is how one would *approach* the study of brain mechanisms. Rather than focusing on regions (Mills et al., 2014) or nodes (e.g., ventral striatum, prefrontal cortex) this approach would examine developmental shifts in the flow of information and output within and across circuits” (Casey et al., 2015). In our opinion, the systemic (DSP) interpretation of adolescent maturation goes beyond this claim, since “flow of information” and “outputs of circuits” are computational terms that have no place in complex dynamic systems. From this perspective, “outputs” and “information” are only understood at a global level, and not at a “circuit” sublevel. Other authors propose a wider perspective in order to develop new scientific paradigms. Thus, Pfeifer and Allen introduced PECANS, a ‘checklist’ to successfully conduct research on adolescence (Pfeifer and Allen, 2016). The ‘n’ in the acronym refers to ‘Neural inferences,’ and they recognize that the association between regions and psychological states are many-to-many. They propose, similarly to Steinberg, a simplification of reality, and concentrate on single regions as ‘markers’ of psychological processes. Considering this self-imposed limitation, this approach could be similar to the dispositional tendency proposed by Anderson.

Apart from this neuropsychological evidence, this modular vision of emotional management is also supported by the well-known psychologist Goleman (1995), in particular the term “amygdala hijacking,” which he proposed. He claims that amygdala takes control when the neocortex has not yet taken a decision. Thanks to the regulatory activity, prefrontal areas act as a modulator of those responses from the amygdala and other limbic areas. Thus, Goleman concludes that the prefrontal cortex is the headquarters for emotional self-control, emotional understanding, and an adequate emotional response. Hence, following the main line of our review, this author follows a clearly modular and analytic interpretation of the mind and brain, which eventually evolves into regulatory. In turn, he defines emotional intelligence as the ability to control impulses and wait for delayed rewards, regulate emotional states, and keep calm when required to think (Goleman, 1995).

In conclusion, the paradigmatic CCA of adolescent development defines a set of modules that are categorized into a dual system, composed of a cognitive-control system and a socioemotional system (Shulman et al., 2016). Both are confronted and ideally the former regulates the latter. Even though these two systems may eventually interact, it is important to note that their functions, natures (as cognitive or emotional), and brain correlates are defined from the beginning of the process. This is the main difference from DSP, where all three factors are dynamic, context-dependent, and rely on the whole system.

### Adolescent Emotional Management and the Systemic Perspective

As explained above, the three main characteristics of the DSP are context dependency, functional temporal coupling of brain areas, and neural reuse. This stands in stark contrast to CCA, which advocates stable structure-function associations. One of the most popular systemic conceptualizations of emotions is offered by Luiz Pessoa. According to Pessoa, emotions, feelings, and cognition are conceptual distinctions, but not different events: there is only one integrated event. Classically, emotions are understood as the body state resulting from a salient stimulus, whereas feelings would be the conscious interpretation of those states (Kandel et al., 2000). According to Pessoa, however, emotion is an ambiguous term that comprises all the elements necessary to build an idea of the environment and ourselves, highlighting our personal situation and influencing behavior (Pessoa, 2010b, 2013). At a psychological level, it is more convenient to understand emotions and feelings as simultaneous phenomena that bidirectionally affect each other, both of which have conscious and unconscious consequences (Pessoa, 2010a). A clear-cut distinction between emotions/feelings and cognition is also problematic. They are clearly integrated in typically human behaviors such as attention (Ernst et al., 2011), stress management (LaBar and Cabeza, 2006; McKlveen et al., 2013; Shansky and Lipps, 2013), memory (Holland and Kensinger, 2010), and decision making (Bechara et al., 1999; Bechara, 2004; Fair et al., 2007), for example. Moreover, ‘emotional’ brain regions can be hardly distinguished from ‘cognitive’ ones, since they are reciprocally connected, and therefore they require each other. A malfunction in one means a failure of both (LeDoux, 2000; Pessoa, 2008; Manfrinatia et al., 2013; McKlveen et al., 2013; Barbey et al., 2014). The distinction between emotions and feelings is conceptually possible, but their boundaries blur at a neural level: both involve highly complex levels of human perception (LeDoux, 2000), and both require the synchronous cooperation of many groups of brain areas (Kober et al., 2009). In conclusion, nearly every human action involves the activity of associative and limbic aspects of the brain, whose neural signal is integrated by reciprocal connections at the very beginning of the process. Emotion regulation-following CCA-entails a distance between cognition and emotion in order to develop cognitive control; however, if there is no gap between them-as suggested by DSP-, it might be better to talk about a “general growth” based on the relationship between cognition and emotion, understood as a conceptual rather than a neuropsychological distinction, from the very beginning. As mentioned above, the main explanatory neurobiological tool of DSP is neural reuse, which entails that focal brain areas can be recruited for diverse tasks, depending on context. Recently, Anderson has reviewed the significance of neural reuse in the organization and development of the brain (Anderson, 2016). According to him, neural reuse is particularly important in neurodevelopment as follows: (i) local regions of the brain are involved in diverse tasks across multiple domains (perception, action, etc); (ii) taking into account interactive differentiation, the set of tasks that each region supports is different from region to region; (iii) early developing regions are reused in more tasks, because they have been available for a longer time; and (iv) the later in development a cognitive function emerges, the more scattered regions are recruited for carrying it out, due to the availability of more useful functional elements. These facts show that brain regions such as the ventral striatum, which mature early in adolescence, are available for tasks beyond emotion-related process. In addition, cognitive functions with a delayed maturation in adolescence, like cognitive control, recruit scattered regions such as an inferior fronto-striatal-thalamic-cerebellar network (Rubia et al., 2007). In the following paragraphs, we will summarize the various tasks in which the main components of the limbic systems are involved.

The circuit of Papez (1973), the first neuroanatomical description of the limbic circuit, has been extensively enriched in recent decades (see, for example, the recent revision by Catani et al., 2013). Relevant research shows that the limbic system, in a wide sense, involves many different brain regions, whose activity is integrated through the synchronization of multiple frequency bands (Pessoa, 2008, 2013). Furthermore, a recent meta-analysis of cortical-subcortical interactions in emotional processing concludes that limbic regions (e.g., amygdala) are strongly connected to cognitive areas, such as the prefrontal cortex (Kober et al., 2009). In the following lines we will summarize the role of the main components of the limbic system (amygdala, nucleus accumbens, and cortical regions), stressing their interaction with cognitive-related areas. From DSP, the integration of the neural information coded by each area does not happen at the end of the process, because there is no process without integration at the very beginning.

The main role of the amygdala consists in valuing the environment with respect to the biological needs of the subject. This includes emotional-affective valuations (Pessoa and Adolphs, 2011). However, the brain can also assign values without recruiting the amygdala (LeDoux, 2013), and this brain area also participates in processes unrelated to emotions (Pessoa, 2013). In general, the signal processed in this complex is modulated by afferents from associative regions of the cortex. In fact, the prefrontal corticoamygdaloid projections are distinctive of humans (Pessoa, 2010b; Pessoa and Adolphs, 2011; Schumann et al., 2011).

The nucleus accumbens is mainly in charge of organizing approach or avoidance behaviors with respect to a stimulus or context (Ernst et al., 2006; Hoebel et al., 2007; Carlezon and Thomas, 2009; Brooks and Berns, 2014). Thus, it is involved in motivation for goal-directed actions (Meredith et al., 2008; Wolf and Ferrario, 2010). However, the accumbens is also involved in value-independent associations (Cerri et al., 2014). At a neuroanatomical level, although this ventral aspect of the striatum is included in the limbic system, the information it processes is integrated by reciprocal projections between the nuclei of the basal ganglia, and also by striatal interneurons (Haber, 2003; Bernacer et al., 2005, 2012; Prensa et al., 2009). The conveyance of the neural information to the thalamus and cortex only happens when it is integrated. In fact, the reciprocal influence between the nucleus accumbens and the dopaminergic ventral tegmental area-located in the midbrain-is modulated by glutamatergic projections reaching the accumbens from the cortex and other subcortical nuclei (Picciotto, 2013).

At a cortical level, the orbitofrontal cortex allows the valuation of a stimulus considering the expectations of the subject and the information processed by the amygdala, accumbens, and different cortical regions (Schoenbaum and Roesch, 2005; Ernst et al., 2006; Padoa-Schioppa and Cai, 2011; McDannald et al., 2012). Its efferent connections are mainly sent to the brainstem (McDannald et al., 2012), amygdala, and nucleus accumbens (Blakemore and Choudhury, 2006), facilitating behavioral flexibility (Cardinal et al., 2002). One of the cortical areas initially related with the limbic system was the cingulate cortex, although it is now known to be mainly related to cognitive aspects such as error monitoring and decision making under uncertainty (Carter, 1998; Goñi et al., 2011; Strang et al., 2011). In order to carry out these processes successfully, it is connected with associative (prefrontal cortex) and limbic (amygdala) structures. Besides this, the anterior cingulate, together with part of the insular cortex, has been reported to contain spindle-von Economo-neurons, which are related to self-awareness (Allman et al., 2012). The insula is another key area for understanding the integration of different modalities of information: the posterior aspects integrate perceptual information, which is in turn combined in the central part with the afferents received from temporal, prefrontal, cingulate, and orbitofrontal cortical areas, as well as from the amygdala and hypothalamus. The third level of integration happens in the most anterior part, usually associated with self-awareness (Craig, 2009; Medford and Critchley, 2010; Sporns, 2011).

Although other limbic-related areas could be mentioned, we believe that the main idea of this section is already justified: even if we accept that there are emotional and cognitive areas in the brain, they are intimately related to each other. In fact, the interaction is even closer at a behavioral level. From a systemic perspective, emotions and cognition are conceptual categorizations, but they are not processed independently in the brain. Therefore, suggesting a cognitive regulation of emotions is not realistic, because they are intrinsically integrated in their own development. Rather, from DSP, emotions are integrated into human behavior. In any case, experimental research on adolescent neurodevelopment from DSP still needs to be boosted. The main empirical tool for demonstrating neural reuse is the functional fingerprinting proposed mainly by Anderson et al. (2013). This is a sort of meta-analytical method that creates a multidimensional vector for each brain region of interest. Each of the dimensions of the vector corresponds to different domains where a task can be categorized, for example: motor learning, motor observation, emotions-happiness, emotions-disgust, and so on. These diversity profiles are intended to reveal all the different task domains in which a particular brain area is involved. In other words, the neurons in that area are reused for as many task types as the plot shows. To the best of our knowledge, no research has applied this method to adolescents. Furthermore, it would be extremely interesting to conduct this kind of study in longitudinal analyses, although the methodological limitations are obvious. Apart from the research field of neurodevelopment, several authors within psychology have stressed the importance of developing models that shift the focus away from the late maturation of cognition after adolescence. For example, Reyna and Farley (2006) state that “contrary to popular wisdom, adolescents see themselves as more vulnerable than adults do, and they typically overestimate important risks.” Moreover, they argue that even though reasoning evolves during and after adolescence, some biases emerge that produce violations of coherence more frequently in adults than in youngsters. In a similar way, using a gambling task, empirical research reveals that adolescents are rational decision makers, since they tend to reject disadvantageous gambles and to accept more favorable options more often than adults (Barkley-Levenson and Galvan, 2014). Finally, it has recently been suggested that peaks in ventral striatum sensibility, which has classically been interpreted as a neural correlate of risk- or sensation-seeking, can be an adaptive neural mechanism to promote healthy behavior in adolescents, such as academic motivation, engagement in hobbies, healthy peer relations, and prosocial behaviors (Telzer, 2016). According to the author, the interpretation of this neurodevelopmental fact may be understood as a source of vulnerability or of opportunity, depending on context (maternal presence, peer influence, etc.).

## Conclusion. Two Accounts of Adolescent Emotional Management: Regulation and Integration

In this manuscript we have reviewed the neuropsychological development of adolescents in two different interpretations of brain functioning, namely componential computational and dynamic systemic. We have shown that the interpretation of the adolescent’s reality is different depending on the model, especially in terms of emotional management. Remarkably, we have argued that the key words that best define emotional management in these models are regulation and integration, respectively. Based on the scientific literature CCA is the predominant view, wherein the emotional-related aspects of the adolescent’s brain escape the regulation of the cognitive regions. Previous research on adolescents’ development from DSP is scarce, so we have based our review on novel models of emotion and neural reuse in this interpretation. According to these models, a particular brain area cannot be associated with a single function, and ‘cognitive’ regions cannot regulate ‘emotional’ areas because their function is integrated from the beginning of the process: although the terms should be conceptually distinguished, there is no emotion without cognition, and vice versa.

The final goal of our review was to show that the global interpretation of adolescent maturation depends on the underlying account of brain functioning. Ultimately, this will affect educational and legal policies to promote the best personal development of adolescents. Before being implemented, the question of whether regulation or integration is the best approach to promoting emotional self-management in adolescents should be considered. In this respect, CCA of the mind and brain and the subsequent interpretation of emotional management as regulation assume that: (i) regulatory and regulated modules are clearly identified, and thus a distance exists between them; (ii) there is a starting point (emotional situation A) and an end point (emotional situation B); (iii) the relationship between the cognitive and the emotional modules is unbalanced, such that the latter automatically impacts on the former in early adolescence, and the former’s goal throughout the process is to end up controlling the latter. With respect to the educational and legal consequences deriving from this account, a detailed description is far beyond the scope of this article. The simplified idea would be that adolescents are in a vulnerable condition due to their imbalanced development. Therefore, legal and educational systems need to build a context within which adolescents are less prone to harming themselves or others. Concerning emotions they are understood as drivers of behavior, and youngsters should focus on being guided by positive emotions within the constraints set by the context.

On the other hand, if brain architecture is viewed under the scope of DSP, emotional management is interpreted as involving the whole system. In this case, emotions are integrated in the whole development of the subject. In our opinion, emotional management from DSP is framed as the growth of the whole person. The assumptions in this case are: (i) there are no brain modules, and mental processes-involving cognition and emotion-correlate with the activity of the brain as a whole; (ii) personal development does not involve a fixed end point, that is, a final emotional state that should be reached: integration of cognition and emotion delimitates a pathway of unrestricted growth, where the important issue for the subject is the process itself, rather than the final point; (iii) in the development of a system, all elements interact with each other from the beginning of the process; therefore, there is no independent transformation of elements.

What are the possible implications of DSP in terms of legal or educational policies? The systemic view and its associated understanding of emotional management, as we have proposed here, emphasize one critical point of adolescence: responsibility. Youngers are not viewed as vulnerable, but as living a unique opportunity to develop their own personal identity. Therefore, the educational system has to help them to seize the opportunity by going beyond the resolution of concrete immediate problems, and aiding the adolescent to contextualize each decision in a continuous growth landscape. From this perspective, emotions give information about how development is taking place: they are something to reflect on, but not drivers of behavior.

The prestigious philosopher of education Richard Stanley Peters states that the final aim of education is to turn adolescents into better men and women, and this is not achieved through mere training in certain cognitive or emotional abilities. The final goal of adolescent education points toward a global interpretation of one’s own life and of the rest of the world, which goes beyond particular skills (Peters, 1966, 1967). Although we have presented the two interpretations of brain architecture and their resulting views of emotional management objectively, we believe that emotional management as integration is more suitable and realistic in accounting for the adolescent’s reality. Following Peters’ argumentation, emotional regulation would then be understood as a particular ability of the person, whereas emotional integration affects the whole person. In our opinion, recent neuroscientific findings demonstrate that DSP is a better model to explain brain anatomy and functioning, since CCA requires more conceptual assumptions. Likewise, integration may be a more adequate framework for emotional management than regulation, since it does not require conceptualizing the fragmented reality of adolescents.

## Author Contributions

JVOS originally conceived the hypothesis of this work. JVOS, JIM, and JB developed the hypothesis and contributed to the preparation of the manuscript.

## Conflict of Interest Statement

The authors declare that the research was conducted in the absence of any commercial or financial relationships that could be construed as a potential conflict of interest.
